# Processing and Modeling of Alginate Hydrogel for Radiologically-Equivalent Biomedical Phantoms

**DOI:** 10.3390/gels12050355

**Published:** 2026-04-23

**Authors:** Olusegun J. Ilegbusi, Godson N. Brako, Chiranjit Maiti, Jihua Gou

**Affiliations:** Department of Mechanical and Aerospace Engineering, University of Central Florida, Orlando, FL 32816, USA; godson.brako@ucf.edu (G.N.B.); chiranjit.maiti@ucf.edu (C.M.); jihua.gou@ucf.edu (J.G.)

**Keywords:** alginate hydrogel, radiologically equivalent phantom, computational fluid dynamics, bubble dispersion, void fraction, Hounsfield Unit, radiological property

## Abstract

The foaming of hydrogels presents a promising strategy for tailoring mechanical and radiological properties to replicate biological soft tissues for biomedical phantom applications. A computational fluid dynamics (CFD) framework is developed to predict void fraction distribution in alginate hydrogel precursor solutions aerated by air injection through a bottom nozzle. The objective is to use the framework for the design of the foaming system to match the desired gas-fraction distribution and radiological property. Seven parametric cases are investigated, varying inlet air velocity, alginate concentration, and surface tension. Results show that higher inlet velocities promote stronger jet penetration and greater gas accumulation, while increasing alginate concentration confines the bubble plume, with quasi-steady gas fractions displaying a non-monotonic trend with concentration. Elevated surface tension yields broader plume coverage and improved gas distribution uniformity at the expense of peak void fraction. The predicted void fractions map to Hounsfield Unit (HU) values of −34 to −103, corresponding to adipose and fatty breast tissue attenuation (−50 to −150 HU). The peak gas fraction at 5.0 wt% alginate yields −307 HU, approaching published experimental CT measurements for the same formulation (−460 to −233 HU).

## 1. Introduction

The incorporation of bubbles into alginate hydrogels has emerged as a critical technique for the development of radiologically equivalent phantoms, surgical rehearsal models, and device verification protocols that require accurate reproduction of both mechanical compliance and radiological response of soft tissues [1,2]. Alginate hydrogels are particularly suitable due to their biocompatibility, mild ionic gelation conditions, and tunable stiffness from sub-kPas to several tens of kPas [3,4]. Despite the promise of foaming alginate hydrogels, empirical process protocols suffer from batch-to-batch variability in void fraction distribution that translates directly to unacceptable scatter in the achieved Hounsfield Unit (HU), and no predictive tool currently exists to guide foaming process parameters toward a prescribed radiological target. The present work addresses this gap by developing a CFD framework that links controllable process inputs directly to spatial void fraction and the resulting HU outcome [1]. The inclusion of air bubbles produces a foam-like structure that reduces density and modulus while tuning radiopacity, enabling fabrication of radiologically equivalent phantom materials [1].

The development of radiologically equivalent biomedical phantoms requires a multi-stage material engineering approach, beginning with identification of the target Hounsfield Unit (HU) range for the tissue of interest, which spans from −50 to −150 HU for adipose tissue, +20 to +40 HU for fibroglandular breast tissue, and −600 to −900 HU for inflated lung parenchyma [1,5,6,7]. Air bubbles are introduced into the hydrogel to reduce the effective density and produce effective target HU based on the linear attenuation mixing rule [1,8]. Qiu et al. [5] demonstrated that alginate-based hydrogel composites can be independently tailored in CT attenuation, ultrasound contrast, and elastic modulus to simulate tissues from adipose to skin. Following bubble dispersion, the structure is locked in place by ionic or photoinitiated crosslinking before significant gel network formation [2]. The final phantom is typically characterized by CT scanning to validate that the achieved HU and mechanical properties fall within acceptable tolerances [5,9,10]. The preparation of alginate hydrogel foams begins with the aim of dissolving sodium alginate powder and SLES 70 in deionized water to form a viscous precursor solution, where the polymer and surfactant concentration directly controls the solution rheology and, consequently, the ability of the matrix to entrap and retain dispersed air bubbles during the foaming process [1,2].

The present study focuses on the use of Computation Fluid Dynamics (CFD) technique for the design of the foaming process. Specifically, CFD is used to model the bubble dispersion stage of the overall process, which determines the spatial void fraction distribution within the hydrogel prior to gelation. This approach directly addresses the challenge of achieving uniform distributions that conventional empirical methods fail to produce reliably [11,12]. Rouhollahi et al. [13] demonstrated that pore size and spatial distribution in freeze-cast chitosan–alginate scaffolds are highly sensitive to processing parameters, reinforcing the importance of CFD-based approaches. The CFD model employed here is first validated against the experimental gas-stirred ladle measurements of Woo et al. [14], as detailed in Section 2.1. The framework is further applied to predict HU values for the 5 wt% alginate formulation of Li et al. [1], enabling direct comparison against their experimental CT data. The significance of this work extends to volumetric 3D bioprinting, where controlled void fraction distribution is critical for perfusable scaffolds [2,15,16,17].

## 2. Results and Discussion

### 2.1. Model Validation

The air–water system was selected as the validation benchmark for several reasons. First, alginate hydrogel precursor solutions at the concentrations studied (0.5–5.0 wt%) are predominantly water by composition [2], with the water content ranging from 95 to 99.5 wt% by definition of the weight fraction. The continuous phase is therefore largely similar to water, making air-water flow a physically representative surrogate for the validation exercise. Second, both the air–water and air–alginate systems are governed by the physical laws expressed by the continuity and Navier–Stokes equations and employ the same interphase closure models. Substituting alginate material properties in place of water modifies the magnitudes of viscous and surface tension forces. Third, the air–water system affords well-controlled experimental measurements of velocity profiles that are not achievable in alginate due to its optical opacity and rapid gelation upon mixing. Furthermore, extensive experimental studies exist for air–water systems, and their data are widely available. However, there is currently a lack of published experimental data on bubble dispersion and void fraction distribution in alginate hydrogel systems, and direct experimental validation of such systems was beyond the scope of the present study. Validation against the gas-stirred ladle experiments of Woo et al. [14]; therefore, constitutes a test of the hydrodynamic framework under conditions that are physically close to the alginate system while remaining experimentally accessible. In addition, indirect validation against the Li et al. [1] CT data for the 5.0 wt% alginate formulation provides evidence that the framework is predictively useful for alginate systems.

**Figure 1:**
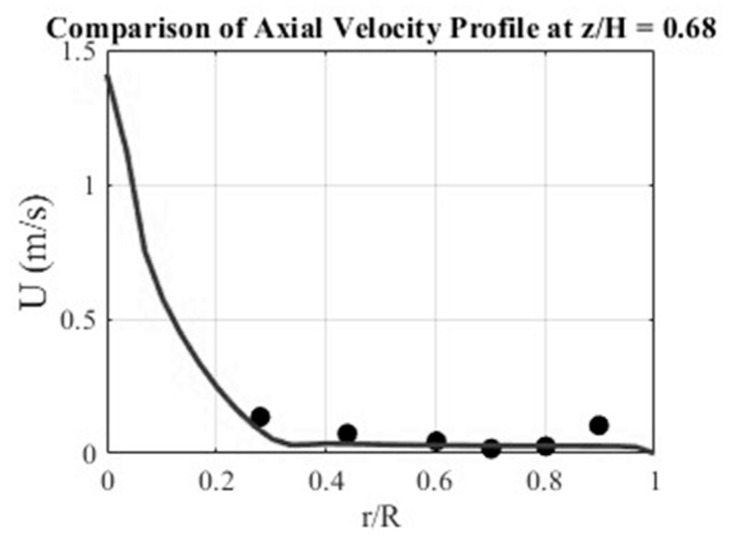
Comparison of simulated and experimental axial velocity profiles at mid-height region (z/H = 0.68).

The computational framework was first validated against air–water experiments before extension to alginate hydrogels. Figure 1 compares the simulated radial velocity profile with the experimental data of Woo et al. [14] at mid-height (z/H = 0.68), yielding a root-mean-square error of 0.042 m/s and a mean absolute error of 0.030 m/s. The curve represents the CFD-predicted axial liquid velocity, and the dots are the experimental measurements of Woo et al. [14]. Here, z is the axial coordinate, and H is the total liquid height. The results confirm that the model adequately reproduces the characteristic radial decay of axial velocity from the centerline to the wall. Similar results were obtained at other locations, but not presented here for brevity.

### 2.2. Air-Alginate Hydrogel Systems

The baseline alginate formulation (Cases A–C) uses properties representative of ~1 wt% calcium-crosslinked sodium alginate (density 1005 kg/m^3^ [4,18], viscosity 0.225 Pa·s [19,20], surface tension 0.040 N/m [3,21,22]), with a constant Newtonian viscosity justified by moderate bulk shear rates (below 100 s^−1^), a bubble Reynolds number of 0.004 confirming the Stokes regime for Schiller–Naumann drag validity [23,24], and an Eotvos number of 4.9 × 10^−4^ confirming spherical bubble shapes [22,25]. At shear rates below 100 s^−1^, the viscosity of 1 wt% alginate deviates by less than 15% from its zero-shear plateau based on published flow curves [19,20], confirming the Newtonian approximation introduces minimal error for the baseline Cases A–C. For the higher-concentration Cases E and G, shear-thinning would locally reduce viscosity near the nozzle where the shear rates are maximum, leading to a slight overestimate of plume confinement. That is expected to be significant only within the relatively small region near the injection nozzle where shear rates are highest. This is a recognized limitation addressed in Section 3.

### 2.3. Velocity Profile

**Figure 2:**
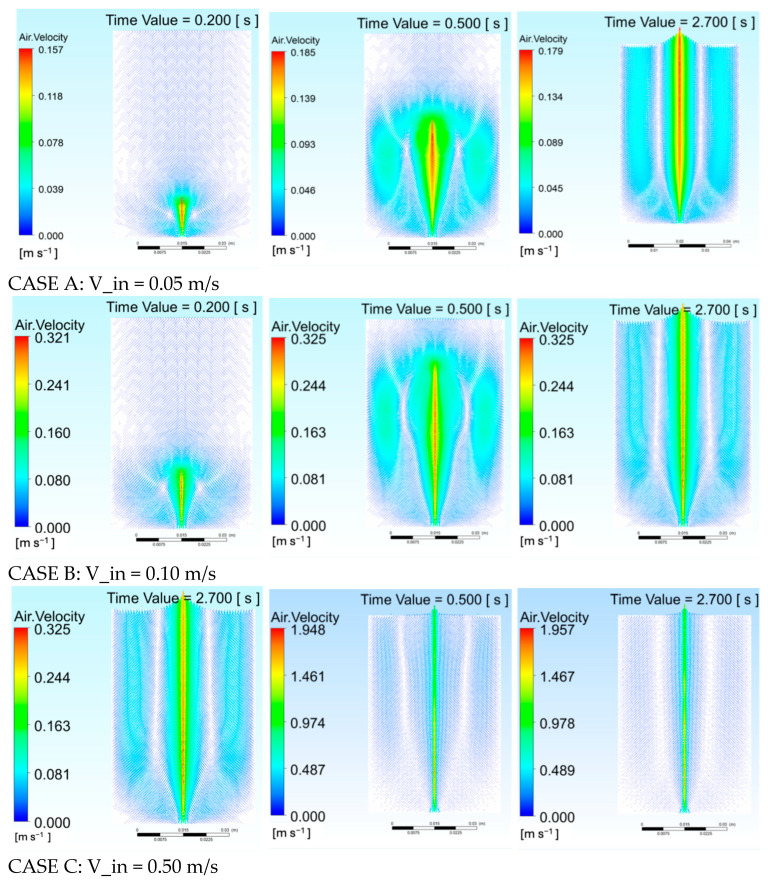
Temporal evolution of velocity magnitude contours for alginate hydrogel at three inlet velocities: Case A (0.05 m/s), Case B (0.10 m/s), Case C (0.50 m/s).

Figure 2 presents velocity contours and vector fields for Cases A–C at t = 0.2, 0.5, and 2.7 s respectively, showing progressively stronger jet penetration with increasing inlet velocity (peak centerline velocity from 0.165 m/s in Case A to over 1.9 m/s in Case C), with all cases producing axisymmetric, vertically elongated plumes whose radial growth is constrained by the high viscosity of the alginate matrix.

### 2.4. Void Fraction Distribution

#### 2.4.1. Influence of Inlet Velocity (Cases A–C)

Inlet velocity governs the momentum imparted to the injected gas, directly controlling bubble rise velocity, plume width, and the rate at which void fraction accumulates within the hydrogel matrix [15,21,22], all of which determine whether the target radiological attenuation is achieved within a clinically relevant fabrication time [1].

**Figure 3:**
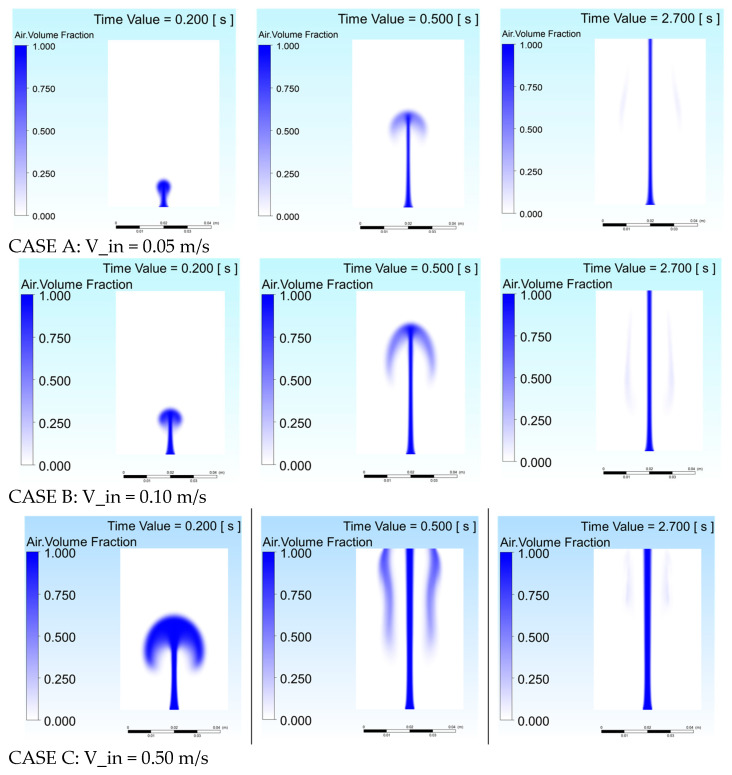
Gas volume fraction contours for alginate hydrogel at different inlet velocities: Case A (0.05 m/s), Case B (0.10 m/s), Case C (0.50 m/s).

Figure 3 presents gas volume fraction contours for Cases A–C corresponding to the time instances presented for velocity vectors of Figure 2, revealing a transition from a narrow, slowly developing gas column at 0.05 m/s to rapid plume development with significant lateral expansion at 0.50 m/s, though hydrogel viscosity and surface tension impose a stabilizing effect that limits dispersion and plume fragmentation.

#### 2.4.2. Influence of Hydrogel Concentration (Cases D and E)

Alginate concentration determines the viscosity of the hydrogel matrix [2,4,19], which controls the resistance to bubble lateral migration and the degree to which buoyancy-driven rise is confined to the central axis [21]. Understanding this relationship is essential for selecting a formulation that balances spatial void fraction uniformity against centerline gas retention to match a target tissue attenuation profile.

**Figure 4:**
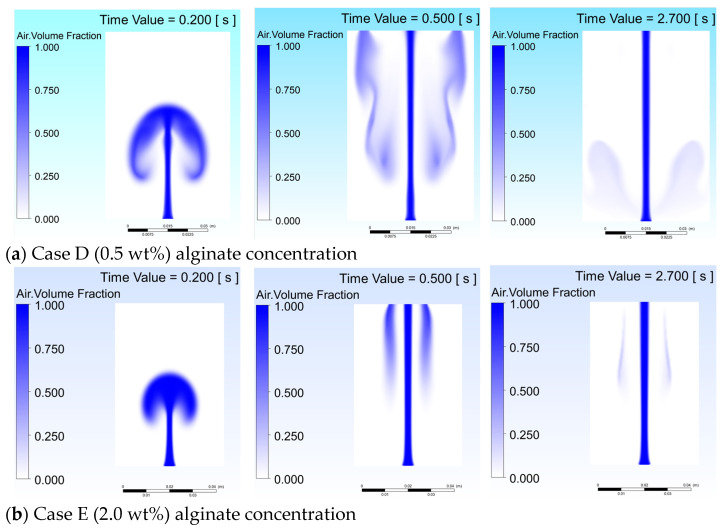
Void fraction evolution comparing (**a**) Case D (0.5 wt%) and (**b**) Case E (2.0 wt%) alginate concentrations.

Figure 4 shows the void fraction evolution for Case D (0.5 wt%, μ = 0.030 Pa·s) and Case E (2.0 wt%, μ = 0.400 Pa·s) at t = 0.2, 0.5, and 2.7 s, where lower viscosity in Case D facilitates early mushroom-shaped plume expansion with well-distributed void fraction and secondary gas pockets [22], while higher viscosity in Case E restricts plume width to less than 20% of domain radius with a laminar column-like structure [2,15]. These results confirm that alginate concentration governs the trade-off between lateral coverage and centerline gas retention.

#### 2.4.3. Influence of Surface Tension (Case F)

Surface tension at the gas–liquid interface governs bubble formation size, coalescence resistance, and the lateral spreading of the rising plume, all of which directly influence the spatial uniformity of void fraction within the hydrogel matrix [3,26,27].

**Figure 5:**
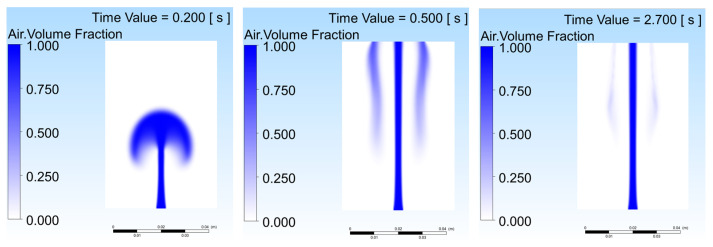
Void fraction evolution in an alginate hydrogel at 0.5 m/s with elevated surface tension (σ = 0.072 N/m).

Figure 5 presents void fraction distributions for Case F (σ = 0.072 N/m) at 0.2, 0.5, and 2.7 s, showing enhanced lateral expansion (plume widths reaching 45% of vessel diameter) with lower peak void fractions (0.55–0.60 versus 0.70+ in baseline Case C), as higher surface tension stabilizes bubble interfaces and resists coalescence [18,21]. While maximum localized gas concentration was reduced, volumetric uniformity improved markedly, consistent with evidence that elevated surface tension fosters more uniform dispersion [2,21,22,26,27].

#### 2.4.4. Influence of High Alginate Concentration (Case G)

At high alginate concentrations, the pronounced increase in matrix viscosity fundamentally alters bubble hydrodynamics by suppressing lateral momentum transfer and promoting axial confinement of the rising plume [2,25], a regime not fully captured by the lower-concentration cases and directly relevant to the 5.0 wt% sodium alginate formulation reported by Li et al. [1] for lung-mimicking phantom fabrication [4,28].

**Figure 6:**
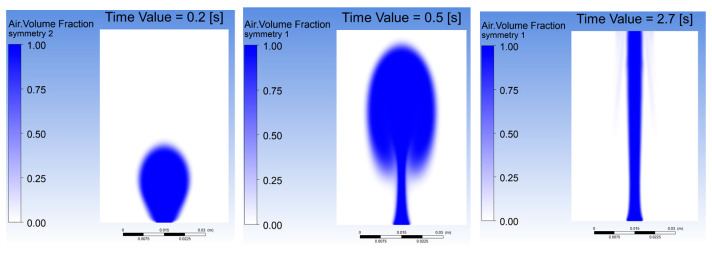
Void fraction contours for Case G (5.0 wt% alginate, Li et al. [1] formulation) at t = 0.2, 0.5, and 2.7 s.

Figure 6 presents void fraction contours for Case G (5.0 wt% alginate, *μ* = 3.500 Pa·s, σ = 0.033 N/m, *v*_in_ = 0.10 m/s) at t = 0.2, 0.5, and 2.7 s, respectively, corresponding to the Li et al. [1] formulation. At t = 0.2 s, an egg-shaped gas pocket forms above the nozzle, confined within the lower 30% of the vessel height. Despite the early injection stage, the high precursor viscosity already suppresses lateral momentum transfer relative to Cases A–F at the same time instant. By t = 0.5 s, coinciding with the peak domain-averaged gas fraction (0.31), the plume has developed a distinctive mushroom morphology: a narrow stem rising from the nozzle feeds a broad cap that has expanded laterally to approximately 60% of the domain radius and vertically to roughly 85% of the vessel height. This transient cap expansion reflects the large gas inventory accumulated at peak injection and is the primary contributor to the high domain-averaged void fraction at this instant. The mushroom shape contrasts with Case E (2.0 wt%), which at comparable times exhibited a more diffuse plume without a clearly defined cap structure. By t = 2.7 s, the cap has collapsed, and the gas has consolidated into a narrow, persistent columnar jet spanning the full vessel height, with the high-concentration core confined to approximately 20% of the domain radius. This quasi-steady structure, characterized by steep radial void fraction gradients and minimal lateral gas penetration, is consistent with the domain-averaged gas fraction stabilizing at 0.10, above Case E (0.06), because the extreme viscosity traps gas centrally and prevents rapid vertical escape, so that injected gas accumulates along the axis rather than dispersing or exiting the domain [2,22,25].

### 2.5. Gas Fraction Evolution and Uniformity

Achieving spatially uniform void fraction distribution is critical in phantom fabrication, as non-uniform gas retention produces heterogeneous Hounsfield Unit (HU) distributions that do not replicate the homogeneous attenuation signature of real lung tissue observed in clinical CT imaging [1,5], making quantitative assessment of both temporal evolution and spatial uniformity essential for process optimization [11,12]. It is emphasized that the CFD simulations model bubble dispersion in the ungelled precursor only and crosslinking is not modeled. The physically relevant pre-gelation window for the CaCO_3_/GDL system at room temperature is approximately 5–10 min, within which all quasi-steady states reported here fall. The physical phantom, therefore, corresponds to the flow state at the time of gelation onset [1].

**Figure 7:**
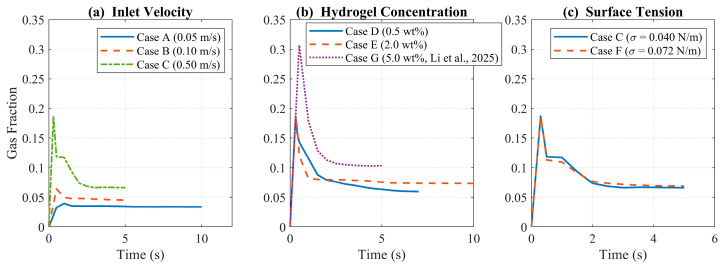
Gas fraction evolution for (**a**) varying inlet velocities (Cases A–C), (**b**) hydrogel concentrations (Cases D, E, and G [1]), and (**c**) surface tension conditions (Cases C and F).

Figure 7 presents the temporal evolution of domain-averaged gas fraction for all seven cases, where Case C and Case F show the highest initial peaks (~0.18) but differ in retention behavior, and Case D achieves the highest sustained gas fraction among Cases A–F (stabilizing at 0.07–0.08) due to reduced viscosity facilitating bubble mobility [4,15,19]. Case E exhibits the most dampened response (peak ~0.10, stabilization ~0.06) from viscous confinement [18,29], while Case A plateaus near 0.03. Case G (5.0 wt%, μ = 3.5 Pa·s) exhibits the highest peak gas fraction of all cases (0.31 at t = 0.5 s), then decays to a quasi-steady value of 0.10. This quasi-steady value is notably above that of Case E despite the substantially higher viscosity, representing a non-monotonic trend with concentration. The physical explanation is that the extreme viscous suppression in Case G confines bubbles into a narrow columnar jet at quasi-steady state (plume width approximately 20% of domain radius), preventing rapid vertical escape, so that gas accumulates centrally and the domain-averaged fraction remains elevated. Collectively, these results demonstrate that inlet velocity, alginate concentration, and surface tension each play distinct roles in balancing bubble stability and volumetric distribution [2,11,21,22].

To quantify spatial homogeneity of air bubbles in the hydrogel, the area-weighted Uniformity Index (UI) of the air phase was computed:

(1) $$\gamma_{a}=1-\frac{\sum_{i=1}^{n}\left|{(\phi }_{i}-{\bar{\phi }}_{a})A_{i}\right|}{2{\bar{\phi }}_{a}\sum_{i=1}^{n}A_{i}}$$

where $A_{i}$ is the area of the facet i, $\phi_{i}$ is the field variable (here, void fraction) at the facet i, and ${\bar{\phi }}_{a}$ is the area-weighted average value over the surface.

**Figure 8:**
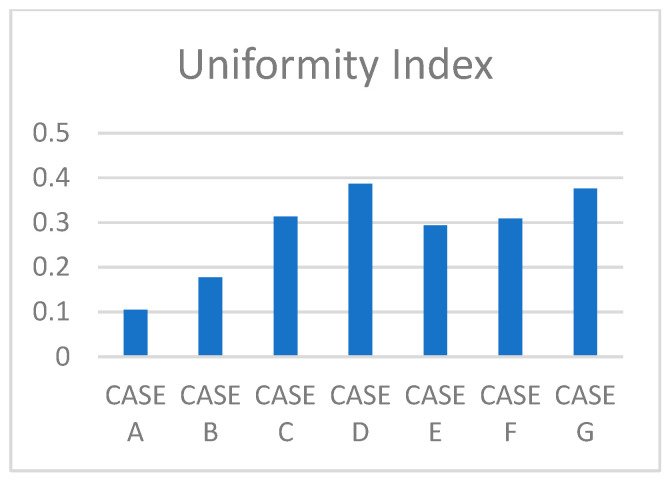
Uniformity Index (UI) for all seven simulation cases: 0.05 m/s, 0.1 m/s, 0.5 m/s, 0.5 wt% hydrogel, 2.0 wt% hydrogel, elevated surface tension (σ = 0.072 N/m), and 5.0 wt% hydrogel (Li et al. [1]).

Figure 8 displays computed UI values across all seven cases investigated at peak gas fraction instances. Case D achieved the highest UI (0.39), followed closely by Case G (0.38), showing that markedly different alginate concentrations can yield similar spatial gas uniformity through fundamentally different physical mechanisms. Although the numerical difference in UI between Case D (0.39) and Case G (0.38) is small, this convergence is physically meaningful. Specifically, low viscosity drives uniformity through free lateral bubble migration, while extreme viscosity achieves comparable uniformity through transient cap expansion at peak gas fraction. This mechanistic contrast is a key finding of the parametric study. Case D achieves uniformity through low hydrogel viscosity, enabling free lateral bubble migration, while Case G achieves comparable uniformity because the transient mushroom-shaped plume at peak gas fraction (t = 0.5 s) fills approximately 60% of the domain radius with a dense, coherent gas cap. Cases C and F follow at 0.31 and 0.30, respectively, while Case E (0.29) and Case B (0.18) exhibit intermediate values. The lowest UI occurred in Case A (0.10), reflecting limited bubble spread at low inlet velocity. These results confirm that flow parameters and material properties must be jointly optimized for spatially consistent gas dispersion [1,2,11].

### 2.6. Radiological Property Estimation

The radiological property represented by the Hounsfield Unit (HU) value of the foam material is estimated from the void fraction using the linear attenuation mixture rule [1]:

(2) $${\mathrm{HU}}_{\mathrm{foam}}=\alpha_{g}\cdot {\mathrm{HU}}_{\mathrm{air}}+\left(1-\alpha_{g}\right)\cdot {\mathrm{HU}}_{\mathrm{hydrogel}}$$

where $\alpha_{g}$ is the volume-averaged gas fraction, ${\mathrm{HU}}_{\mathrm{air}}$ = −1000, and ${\mathrm{HU}}_{\mathrm{hydrogel}}$ ≈ 0 for water-equivalent alginate hydrogels [1,2].

**Figure 9:**
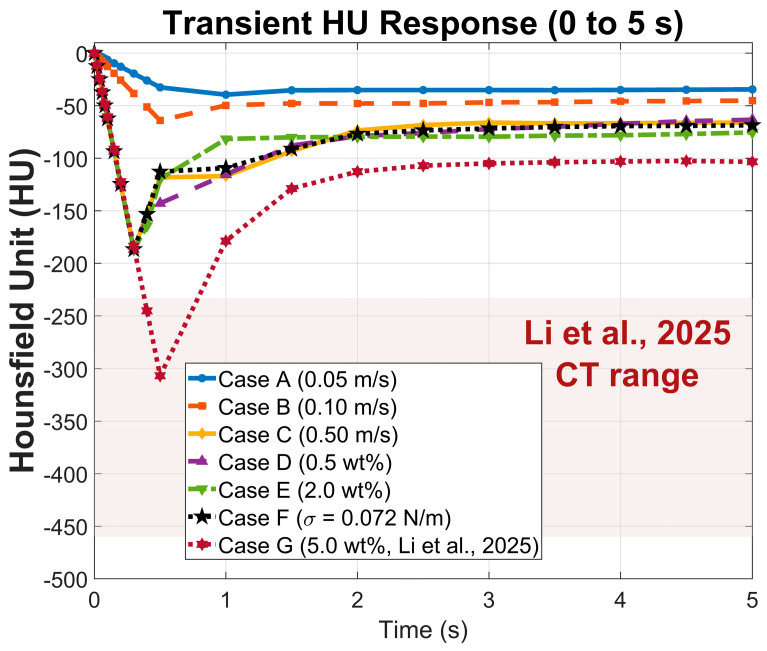
Transient HU response (0 to 5 s) for all seven simulation cases [1], computed from domain-averaged gas fractions using the linear attenuation mixture rule (Equation (5)).

Figure 9 presents the transient HU evolution for all seven cases over the first 5 s, where all cases originate at 0 HU and decrease as gas accumulates, with Case C (high inlet velocity, 0.50 m/s) exhibiting the steepest initial decline to −187 HU before recovering to −66 HU as excess gas escapes. Cases A (low inlet velocity, 0.05 m/s) and B (medium inlet velocity, 0.10 m/s) show monotonic reductions plateauing at −34 and −45 HU respectively, while Cases D (0.5 wt% alginate), E (2.0 wt% alginate), and F (elevated surface tension, σ = 0.072 N/m) stabilize between −59 and −73 HU, reflecting the distinct influences of viscosity and surface tension on bubble retention [19,20,26]. Case G (5.0 wt% alginate) exhibits the deepest transient HU drop, reaching −307 HU at peak gas fraction (αg = 0.31) before recovering to a quasi-steady value of −103 HU (αg = 0.10). The quasi-steady HU values across all cases (−34 to −103 HU) span the range characteristic of adipose and fatty breast tissue (−50 to −150 HU) [1,5,7], indicating suitability for mimicking these soft tissue types [1,2]. The Case G peak HU of −307 approaches the experimental CT measurements reported by Li et al. [1] for the same 5 wt% alginate formulation (−460 to −233 HU), providing some validation to our predictions. However, the predicted quasi-steady value of −103 HU falls above that experimental range, suggesting that the foam microstructure is arrested during the transient high-gas-fraction phase, consistent with rapid gelation kinetics of the CaCO_3_/GDL system. This agreement is interpreted as qualitative because gelation kinetics are not coupled to the flow solver. Coupling a gelation kinetics model with our existing model will be pursued as a priority for future work. Extension to lung parenchyma (with HU range −600 to −900) [6,9] will necessitate void fractions exceeding 0.60. Achieving such high gas fraction values will require proper redesign of our manufacturing system for higher gas injection velocities and high gas retention, use of multi-nozzle configurations, and perhaps introduction of additional mixing mechanisms such as mechanical agitation to promote better uniformity of gas fraction distribution [1,28].

## 3. Conclusions

An Eulerian two-fluid CFD framework, validated against air–water experimental data and applied predictively to alginate hydrogel systems, is used to predict bubble dispersion in the foaming of alginate hydrogels for the development of radiologically equivalent biological phantoms. The framework was first validated against an air–water experimental benchmark and subsequently extended to the alginate systems by utilizing experimentally derived material properties.

Seven parametric cases were investigated, indicating that inlet velocity, hydrogel concentration, and surface tension exert distinct, yet interconnected influences on void fraction distribution and the resulting radiological properties. Higher inlet velocities promote stronger jet penetration and greater initial gas accumulation but delay stable plateaus. Lower alginate concentrations enable broader lateral plume expansion and higher spatial uniformity, while higher concentrations confine the plume to the central axis and produce a non-monotonic gas retention trend driven by viscous trapping. Elevated surface tension stabilizes bubble interfaces, yielding wider radial spread and more uniform plume morphology at the expense of peak void fraction.

The quasi-steady void fractions across all cases map to HU values of −34 to −103, spanning the CT attenuation range characteristic of adipose and fatty breast tissue (−50 to −150 HU) [1,5,7]. At the highest concentration investigated (5.0 wt%), the peak gas fraction of 0.31 yields −307 HU, approaching the experimental CT measurements of Li et al. [1] for the same formulation (−460 to −233 HU), providing validation of the framework for high-concentration alginate systems. Extension to lung parenchyma (−600 to −900 HU) would require void fractions exceeding 0.60, which can only be achieved through a redesign of the injection system to enable higher gas velocities, multi-nozzle configurations, or additional mixing mechanisms [1,28].

Combined with the ionic crosslinking protocol used to stabilize the foam structure post-aeration, these results establish a computational-to-fabrication pathway for producing radiologically equivalent biological phantoms with prescribed radiological properties. Several simplifying assumptions were made in the present CFD framework that may require relaxation in future work.

1. Constant Newtonian viscosity: The continuous phase was treated as a Newtonian fluid with a fixed viscosity for each case. It is desirable to explore concentration-dependent and shear-rate-dependent viscosity models to capture the shear-thinning behavior reported for alginate precursor solutions [19].
2. Decoupled crosslinking kinetics: Gelation was not modeled during the bubble dispersion stage. Coupling crosslinking kinetics with the multiphase flow solver would enable prediction of the evolving gel network and its effect on bubble arrest and final foam morphology [28].
3. Single-nozzle, monodisperse injection: A single inlet with a fixed bubble diameter was used to develop the CFD framework. Multi-nozzle injection strategies and population balance models [21] could be explored to extend the framework to polydisperse bubble size distributions and more complex injection geometries.
4. Constant surface tension: Interfacial tension was held fixed for each case. Incorporating surfactant transport and adsorption kinetics [11] would capture the dynamic interfacial effects that govern bubble coalescence and breakup in real foaming processes.

Each 2D axisymmetric simulation required approximately 2–4 h of wall-clock time on a 20-core workstation (18,486 elements, 1 × 10^−3^ s time step, 10 s simulated time). Extension to 3D or inclusion of population balance models would increase cost by 1–2 orders of magnitude, motivating the use of 2D axisymmetric geometry as a computationally practical design tool.

The above simplifications notwithstanding, the CFD framework has demonstrated the capability to predict and control void fraction distributions in alginate hydrogel foams. By linking process parameters directly to radiological outcomes through the linear attenuation mixture rule, the framework offers quantitative guidance for selecting fabrication conditions to achieve target HU values, thereby reducing reliance on empirical trial and error and accelerating the design-to-fabrication cycle for radiologically equivalent biomedical phantoms.

## 4. Materials and Methods

### 4.1. Advanced Manufacturing Framework

**Figure 10:**
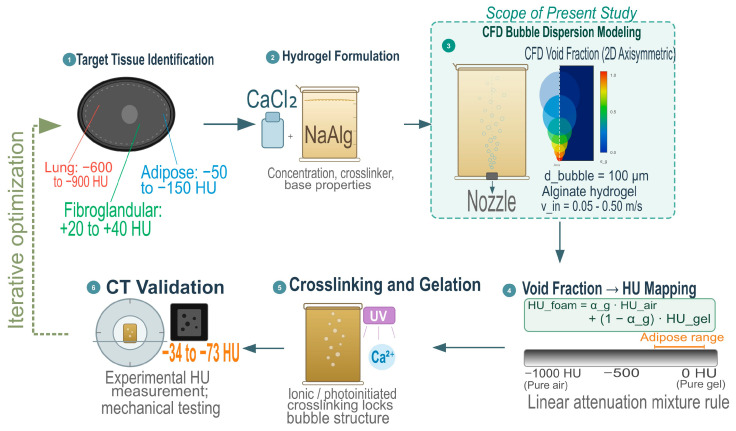
Schematic sketch of the research workflow for biomimetic alginate hydrogel phantom development. (1) Target tissue identification from CT imaging with characteristic HU. (2) Alginate hydrogel formulation from sodium alginate and calcium chloride crosslinker. (3) CFD bubble dispersion modeling (scope of the present study). (4) Mapping of predicted void fractions to Hounsfield Units via the linear attenuation mixture rule. (5) Ionic or photoinitiated crosslinking. (6) CT validation of the gelled phantom against target tissue HU values.

The fabrication of the radiologically equivalent phantom follows the six-stage workflow illustrated in Figure 10. First, the target tissue is identified and its characteristic HU range established from clinical CT data, which can span several hundred Hounsfield Units depending on the tissue of interest, such as adipose tissue (−50 to −150 HU), fibroglandular breast tissue (+20 to +40 HU), or inflated lung parenchyma (−600 to −900 HU) [1,5,6,7]. A hydrogel precursor solution is then prepared by dissolving sodium alginate (NaAlg) in deionized water at a prescribed concentration, with polymer loading selected to achieve the solution rheology necessary for controlled bubble retention [1,2]. Air is subsequently injected into the precursor through a bottom nozzle under conditions informed by the CFD framework developed in the present study, which predicts the spatio-temporal void fraction distribution as a function of inlet velocity, bubble diameter, and alginate concentration. The resulting void fraction is then mapped to an effective HU value via the linear attenuation mixture rule, enabling direct comparison against the target tissue signature [1]. Once the desired void fraction distribution is achieved, the foam structure is permanently stabilized by ionic crosslinking with calcium chloride or photoinitiated curing, which rapidly gels the alginate network and arrests bubble migration before significant coalescence can occur [2,30]. The final phantom is then sectioned and characterized by CT scanning and mechanical testing to validate that the achieved HU and structural properties fall within acceptable tolerances of the target tissue [5,9,10].

### 4.2. Hydrogel Formulation and Synthesis

Alginate hydrogel foams were synthesized following a previously reported method [1] via an internal ionic gelation and air-foaming approach using sodium alginate (product no. W201502), calcium carbonate (CaCO_3_; product no. 795445, MW = 100.09 g·mol^−1^), and glucono-δ-lactone (GDL; product no. G4750, MW = 178.14 g·mol^−1^), all obtained from Sigma-Aldrich (St. Louis, MO, USA). Sodium lauryl ether sulfate (SLES, 70 wt%) was purchased from Renowned Trading LLC (Cypress, TX, USA) and used as a surfactant. Deionized (DI) water was used throughout all stages of foam preparation.

In a typical procedure, sodium alginate and SLES-70 were dissolved in DI water at room temperature under continuous mechanical stirring at 1000 rpm until a homogeneous solution was obtained. The alginate concentration in the precursor solution was varied from 0.5 to 5.0 wt% depending on the target formulation (see Table 1), as polymer loading directly governs solution viscosity, surface tension, and the resulting capacity of the matrix to entrap and retain dispersed air bubbles. The volumetric fraction of SLES-70 was maintained at 2% for foam samples, where the surfactant served to stabilize air incorporation during mechanical agitation, thereby enabling the formation of a porous hydrogel foam architecture. Calcium ions required for alginate crosslinking were generated in situ through the controlled reaction of CaCO_3_ with GDL. The Ca^2+^ to alginate carboxylate (–COO^−^) molar ratio was maintained at 0.18 across all formulations to ensure effective gelation while preserving foam integrity. Simultaneously, a CaCO_3_:GDL molar ratio of 0.5 was employed to achieve gradual acidification and sustained Ca^2+^ release, thereby maintaining the system near neutral pH during gel formation.

For foam generation, a lidded beaker with a total volume of 140 mL was used to regulate the solution-to-air mixing ratio. Based on volumetric considerations, 50 mL of the alginate/SLES solution was introduced into the beaker, a volume specifically selected to allow sufficient air incorporation during mixing. SLES-70 was first added to the alginate solution and stirred until fully dispersed. Subsequently, an aqueous suspension of CaCO_3_ was introduced and mixed thoroughly until a uniform white dispersion was observed. Finally, the GDL solution was added to initiate internal gelation, and mechanical stirring was continued throughout the process. For samples prepared with a 50 mL precursor volume, mixing was terminated once no further increase in the overall foam volume was observed, indicating completion of air incorporation and the onset of sufficient network formation to stabilize the hydrogel foam structure.

The molar ratios (Ca^2+^:–COO^−^ = 0.18, CaCO_3_:GDL = 0.5) are standardized across all formulations. Alginate concentration is the primary variable parameter controlling precursor rheology. The specific case-by-case material properties used in the computational simulations are listed in Table 1. Case G employs the 5 wt% alginate formulation described above and serves as the validation case against the experimental CT measurements reported by Li et al. [1].

### 4.3. Geometry and Meshing for Computational Modeling

The computational domain represents a standard 100 mL laboratory beaker, as shown in step 3 of Figure 10. A two-dimensional half-section of the cylindrical container was modeled by exploiting its axial symmetry. The domain extends 23.4 mm radially and 70.0 mm axially, with a symmetry boundary along the centerline, yielding an effective internal diameter of 46.8 mm. In the computational domain, r denotes the radial coordinate measured from the axis of symmetry, R = 23.4 mm is the maximum domain radius, and z denotes the axial coordinate measured upward from the bottom boundary where the inlet nozzle is located. A circular gas inlet of 4 mm diameter was positioned centrally at the lower boundary for dispersed air phase injection.

A grid independence study with three progressively refined meshes (0.5, 0.3, and 0.2 mm element sizes containing 8428, 18,486, and 41,418 elements, respectively) confirmed that velocity profiles differed by less than 1% between the two finest meshes; the 0.3 mm mesh (18,486 elements) was therefore selected for all subsequent simulations.

### 4.4. Governing Equations

#### 4.4.1. Flow Field Equations

The air-hydrogel system is modeled with a two-fluid Eulerian framework in which each phase occupies a volume fraction, and both phases share a common pressure field. Mass conservation for phase i (gas g or liquid l) is

(3) $$\frac{\partial }{\partial t}(\alpha_{i}\rho_{i})+\nabla \;\cdot \;(\alpha_{i}\rho_{i}u_{i})=0$$

where *α*_i_ is the local volume fraction, *ρ*_i_ is the density, and *u*_i_ is the velocity of phase i. Momentum conservation is expressed by the Navier–Stokes equation for each phase, thus

(4) $$\frac{\partial }{\partial t}(\alpha_{i}\rho_{i}u_{i})+\nabla \;\cdot \;(\alpha_{i}\rho_{i}u_{i}u_{i})=-\alpha_{i}\nabla p+\nabla \;\cdot \;(\alpha_{i}\tau_{i})+\alpha_{i}\rho_{i}g+M_{il}$$

where *p* is the shared pressure, *g* is gravitational acceleration, and *M*_il_ is the interphase momentum exchange. The drag component is evaluated with the Schiller–Naumann correlation [31].

#### 4.4.2. Turbulence Model

The injection of air through the bottom nozzle generates a buoyancy-driven plume that induces recirculating flow patterns within the vessel, and at the inlet velocities considered in this study (0.05 to 0.50 m/s), the resulting Reynolds numbers at the nozzle exceed the laminar–turbulent transition threshold, necessitating a turbulence closure [22,31].

Turbulence was modeled with the Shear Stress Transport (SST) k-omega formulation of Menter to solve independent k and ω transport equations for each phase, as the gas volume fraction in the plume core can reach locally elevated values where algebraic dispersed-phase turbulence closures may inadequately capture the turbulent kinetic energy generated by the rising bubble swarm [22,31,32,33]. The SST blending function combines the near-wall k-omega behavior with the free-stream k-epsilon limit. The turbulent viscosity of the phase i is given by

(5) $$\mu_{t,i}=\rho_{i}\;a_{1}\;\frac{k_{i}}{max\left(a_{1}\;\omega_{i},\;\;S_{i}F_{2}\right)}$$

where *a*_1_ is a model constant, *S*_*i*_ is the strain-rate magnitude of phase *i*, and *F*_2_ is the SST limiter. Interphase turbulent dispersion is handled implicitly through the per-phase solution.

### 4.5. Boundary Conditions and Parametric Cases

The left boundary coincided with the axis of symmetry, imposing zero normal velocity and zero normal gradients. Air was injected through a 4 mm orifice at the base, modeled as a velocity inlet with a gas volume fraction of unity and a monodisperse bubble diameter of 100 μm, consistent with reported hydrogel foam pore sizes of 10–1000 μm [30]. The side walls were treated as no-slip boundaries, while the top boundary was specified as a pressure outlet at zero-gauge pressure. The domain was initially filled with a quiescent, bubble-free liquid. Seven parametric cases were examined to assess the effects of inlet velocity, hydrogel concentration, and surface tension on bubble dispersion (Table 1), using a baseline crosslinked sodium alginate formulation with density 1005 kg/m^3^, viscosity 0.225 Pa·s, and surface tension 0.040 N/m, consistent with experimental measurements for moderately concentrated alginate gels [18,22]. It is acknowledged that recent studies have reported non-Newtonian (shear-thinning) behavior in alginate hydrogel precursor solutions; however, in the present work, a constant Newtonian viscosity was assumed for each case, consistent with the treatment adopted in previous studies [21,25].

### 4.6. Computational Procedure

Transient simulations were performed with ANSYS Fluent 2023 R1 (ANSYS Inc., Canonsburg, PA, USA) using the pressure-based Eulerian multiphase solver with Phase-Coupled SIMPLE pressure–velocity coupling, second-order spatial discretization for momentum and turbulence, and first-order upwind for the volume-fraction equation. A constant time step of 1 × 10^−3^ s maintained a Courant number below unity over 10 s of simulated pre-gelation time [2], with convergence criteria of 10^−4^ for continuity/momentum and 10^−6^ for phase-fraction/turbulence residuals following [31].

## Figures and Tables

**Table 1:** Simulation case definitions.

Case	Parameter	Inlet Velocity [m/s]	Viscosity [kg/m∙s]	Surface Tension Coefficient, σ	Description
A	Inlet velocity	0.05	0.225	0.040	Low velocity
B	Inlet velocity	0.10	0.225	0.040	Medium velocity
C	Inlet velocity	0.50	0.225	0.040	High velocity
D	Concentration	0.50	0.030	0.050	0.5 wt% alginate
E	Concentration	0.50	0.400	0.036	2.0 wt% alginate
F	Surface tension	0.50	0.225	0.072	Elevated σ
G	Concentration	0.50	3.500	0.033	5.0 wt% alginate (Li et al. [1])

## Data Availability

The original contributions presented in this study are included in the article. Further inquiries can be directed to the corresponding author.

## References

[1] Li X., Gou J., Santhanam A.P., Maiti C., Ilegbusi O.J. (2025). Tissue Mimicking Hydrogel Foam Materials with Mechanical and Radiological Properties Equivalent to Human Lung. Sci. Rep..

[2] Lee K.Y., Mooney D.J. (2012). Alginate: Properties and Biomedical Applications. Prog. Polym. Sci..

[3] Takagi S., Matsumoto Y. (2011). Surfactant Effects on Bubble Motion and Bubbly Flows. Annu. Rev. Fluid Mech..

[4] Mancini M., Moresi M., Sappino F. (1996). Rheological Behaviour of Aqueous Dispersions of Algal Sodium Alginates. J. Food Eng..

[5] Qiu H., Nazarenus J., Egeler B., Thode T., Osman F., Osmonov D., Bahr J., Kaps S., Siebert F.-A., Koch R. (2024). Hydrogel System with Independent Tailoring of Mechanics, CT, and US Contrasts for Affordable Medical Phantoms. ACS Mater. Lett..

[6] Hop J.F., Dudurych I., Stams T.R.G., de Bock G.H., Vliegenthart R., Greuter M.J.W. (2025). Towards a More Realistic Anthropomorphic Chest Phantom Using 3D-Printed and Cork-Integrated Components. Med. Phys..

[7] He Y., Liu Y., Dyer B.A., Boone J.M., Liu S., Chen T., Zheng F., Zhu Y., Sun Y., Rong Y. (2019). 3D-Printed Breast Phantom for Multi-Purpose and Multi-Modality Imaging. Quant. Imaging Med. Surg..

[8] Schneider W., Bortfeld T., Schlegel W. (2000). Correlation between CT Numbers and Tissue Parameters Needed for Monte Carlo Simulations of Clinical Dose Distributions. Phys. Med. Biol..

[9] Hong D., Lee S., Kim G.B., Lee S.M., Kim N., Seo J.B. (2020). Development of a CT Imaging Phantom of Anthromorphic Lung Using Fused Deposition Modeling 3D Printing. Medicine.

[10] Mei K., Pasyar P., Geagan M., Liu L.P., Shapira N., Gang G.J., Stayman J.W., Noël P.B. (2023). Design and Fabrication of 3D-Printed Patient-Specific Soft Tissue and Bone Phantoms for CT Imaging. Sci. Rep..

[11] Dhotre M.T., Ekambara K., Joshi J.B. (2004). CFD Simulation of Sparger Design and Height to Diameter Ratio on Gas Hold-Up Profiles in Bubble Column Reactors. Exp. Therm. Fluid Sci..

[12] Besagni G., Varallo N., Mereu R. (2023). Computational Fluid Dynamics Modelling of Two-Phase Bubble Columns: A Comprehensive Review. Fluids.

[13] Rouhollahi A., Ilegbusi O., Florczyk S., Uludağ K. (2020). Effect of Mold Geometry on Pore Size in Freeze-Cast Chitosan-Alginate Scaffolds for Tissue Engineering. Ann. Biomed. Eng..

[14] Woo J.S., Szekely J., Castillejos A.H., Brimacombe J.K. (1990). A Study on the Mathematical Modeling of Turbulent Recirculating Flows in Gas-Stirred Ladles. Metall. Trans. B.

[15] Delnoij E., Lammers F.A., Kuipers J.A.M., van Swaaij W.P.M. (1997). Dynamic Simulation of Dispersed Gas–Liquid Two-Phase Flow Using a Discrete Bubble Model. Chem. Eng. Sci..

[16] Thiele J., Ma Y., Bruekers S.M.C., Ma S., Huck W.T.S. (2014). Designer Hydrogels for Cell Cultures: A Materials Selection Guide. Adv. Mater..

[17] Murphy S.V., Atala A. (2014). 3D Bioprinting of Tissues and Organs. Nat. Biotechnol..

[18] Paul M., Freudig B., Müller-Kirchenbauer J., Heise S., Kraume M. (2022). Bubble Size Distribution and Gas Holdup in Bubble Columns Employing Non-Newtonian Liquids: A CFD Study. Can. J. Chem. Eng..

[19] Charbonier F.W., Indana D., Chaudhuri O. (2021). Tuning Viscoelasticity in Alginate Hydrogels for 3D Cell Culture Studies. Curr. Protoc..

[20] Cuomo F., Cofelice M., Lopez F. (2019). Rheological Characterisation of Hydrogels from Alginate-Based Nanodispersion. Polymers.

[21] Wang T., Wang J., Jin Y. (2006). A CFD–PBM Coupled Model for Gas–Liquid Flows. AIChE J..

[22] Besagni G., Inzoli F., Ziegenhein T. (2018). Two-Phase Bubble Columns: A Comprehensive Review. ChemEngineering.

[23] Schiller L., Naumann A. (1933). Uber die grundlegenden Berechnungen bei der Schwerkraftaufbereitung. Z. Des Vereines Dtsch. Ingenieure.

[24] Ishii M., Zuber N. (1979). Drag Coefficient and Relative Velocity in Bubbly, Droplet or Particulate Flows. AIChE J..

[25] Chhabra R.P. (2006). Bubbles, Drops, and Particles in Non-Newtonian Fluids.

[26] Takagi S., Ogasawara T., Matsumoto Y. (2008). The effects of surfactant on the multiscale structure of bubbly flows. Phil. Trans. R. Soc. A..

[27] Zenit R., Feng J.J. (2018). Hydrodynamic Interactions Among Bubbles, Drops, and Particles in Non-Newtonian Liquids. Annu. Rev. Fluid Mech..

[28] Malektaj H., Drozdov A.D., Christiansen J.D. (2023). Mechanical Properties of Alginate Hydrogels Cross-Linked with Multivalent Cations. Polymers.

[29] Mørch Y.A., Donati I., Strand B.L., Skjåk-Bræk G. (2006). Effect of Ca2+, Ba2+, and Sr2+ on Alginate Microbeads. Biomacromolecules.

[30] Djemaa I.B., Auguste S., Drenckhan-Andreatta W., Andrieux S. (2021). Hydrogel Foams from Liquid Foam Templates: Properties and Optimisation. Adv. Colloid Interface Sci..

[31] ANSYS Inc (2022). ANSYS Fluent Theory Guide.

[32] Menter F.R. (1994). Two-Equation Eddy-Viscosity Turbulence Models for Engineering Applications. AIAA J..

[33] Menter F.R., Kuntz M., Langtry R., Hanjalic K., Nagano Y., Tummers M. (2003). Ten Years of Industrial Experience with the SST Turbulence Model. Turbulence, Heat and Mass Transfer 4.

